# Canola Oil based Poly(ester–ether–amide–urethane) Nanocomposite and Its Anti-Corrosive Coatings

**DOI:** 10.3390/polym13193325

**Published:** 2021-09-28

**Authors:** Manawwer Alam, Mohammad Altaf, Naushad Ahmad

**Affiliations:** Department of Chemistry, College of Science, King Saud University, P.O. Box 2455, Riyadh 11451, Saudi Arabia; maltaf@ksu.edu.sa (M.A.); anaushad@ksu.edu.sa (N.A.)

**Keywords:** canola oil, coatings, fumed silica, corrosion, nanocomposite

## Abstract

The environmental and health hazards associated with petro-based chemicals have motivated the researchers to replace them partially or wholly with renewable resource-based polymers. Vegetable oils serve as an excellent alternative to this end as they are cost effective, eco-friendly, easily available and rich with functional groups amenable to chemical reactions. The aim of the research work is to prepare Canola oil [CANO] derived poly (ester–ether–amide–urethane) (CPEEUA) nanocomposite coating material using N,N-bis (2-hydroxyethyl) fatty amide [CFA] obtained from CANO, Lactic acid [LA], and reinforced with Fumed Silica [FS]. CPEEUA was obtained by esterification, etherification, and urethanation reactions and its structure was confirmed from FTIR and NMR spectral analyses. CPEEUA/FS coatings were found to be scratch resistant, flexible, well-adhered to mild steel panels, and hydrophobic with 2.0–2.5 kg scratch hardness, 150lb/inch impact resistance and >90° contact angle value. They exhibited good corrosion protection in 3.5 wt% NaCl solution as investigated by Potentiodynamic Polarization and Electrochemical Impedance tests. CPEEUA coatings are safe for usage up to 200 °C.

## 1. Introduction

Thermosetting resins are an inevitable part of the coatings industry. As these are derived from petrochemicals, their toxicity, nonbiodegradability, and volatility of starting materials are of prime health, environmental, and safety concerns, and require immediate attention. An alternative greener solution has been found in the development of biobased resins. The latter resins are partially or completely derived from biological sources such as starch, cellulose, lignin, furan, rosin, and vegetable oils [VO]. They are eco-friendly, cheaper, and biodegradable and offer a completely viable alternative to meet strict environmental regulations, reduce consumption of finite petrochemicals, and are competitive in price and performance to existing petrobased thermosetting resins [1,2,3].

Vegetable oils [VO] have established themselves as workhorses of coatings industry. VO such as Linseed, Karanj, Corn, Castor, Rapeseed, Sunflower, Canola, and many others have undergone chemical transformations producing epoxies, polyesteramide [PEA], alkyd, urethane, and others. As these derivatives often lack physico-mechanical strength and adequate corrosion-protective performance, these are tailormade further as organic-inorganic hybrid and nanocomposite coatings, for advanced applications, through reinforcement by nano modifiers [4,5,6,7]^.^ Canola oil [CANO] has high content of oleic acid. It has been used as a plasticizer for poly(3-hydroxybutyrate) and starch films, as lubricant, in production of polyurethanes and polyhydroxyalkanoates, and biodiesel, apart from being used for edible purposes [7,8,9,10,11,12,13,14,15]. The utilization of CANO as protective coatings is not very abundant [16,17,18]. In this study, CANO was selected as a raw material, transformed into diol fatty amide, to prepare CANO based poly (ester–ether–amide–urethane) nanocomposite coatings using lactic acid [LA] for esterification, fumed silica [FS] as nanofiller, and toluene–2,4-diisocyanate [TDI] as curing agent.

LA, 2-hydroxy propanoic acid, can be artificially synthesized or obtained from natural sources. It has a hydroxyl group adjacent to carboxyl group, thus this one molecule can render two different functional groups very suitable to introduce ester and ether linkages in any polymer. It is considered as a leading candidate in biology and medicine. LA has been used in coatings, and serves as a starting material of poly (lactic acid) that is used in packaging [19,20,21,22].

FS, obtained by high temperature hydrolysis of silicon chloride, has been used as nanofiller in epoxy, polyurethane, polyesteramide nanocomposite coatings. It improves thermal stability, wear, and chemical resistance, as well as scratch hardness of coatings [23,24,25,26,27,28]. Linseed, Castor, Corn, and Jatropha oil-based nanocomposite coatings have been prepared using FS with drastically improved properties compared to the pristine coatings [29,30,31,32,33,34]. 

In the present work, CANO was transformed chemically into *N*,*N*′-bis-2-hydroxyl ethyl CANO amide [CFA], by amidation. CFA was treated with LA, producing CANO-based poly (ester ether) amide [CPEEA]. Generally, VO based PEA coatings, obtained by a chemical reaction between a fatty amide diol and a difunctional acid, lack superior mechanical strength and cannot withstand an alkaline medium; i.e., their corrosion protection performance in an alkaline medium is poor [35,36]. Therefore, PEA coatings can be modified by introducing ether linkages in their backbone, which is accomplished by: (i) synthesis of polyesteramide resin by reaction of hydroxyl functional group of VO amide diol with an acid or anhydride, in the first step; (ii) the introduction of ether groups is carried out by chemical reaction with another hydroxyl bearing compound, in the second step, that involves an additional step of modification, i.e., etherification; (iii) followed by curing with an isocyanate or melamine formaldehyde resin [37,38,39,40,41,42,43]. Using LA is advantageous because it reduces this step of ether modification as LA bears a hydroxyl and carboxyl group in same molecule that facilitates producing poly (ether–ester–amide–urethane). 

The structure elucidation, morphology and thermal stability of the synthesized material were studied by FTIR, NMR, XRD, TGA and DSC, and corrosion protection performance was studied by PDP and EIS. The approach provides an alternative pathway for utilization of CANO to develop biobased thermoset coatings for corrosion protection. The main raw materials used are CANO and LA that are naturally available, and no literature report is available on development of poly (ester–ether–amide–urethane) coatings from these as raw materials. The reaction conditions were not very severe and curing of coatings took place at an ambient temperature. In the long run, such VO based thermoset coatings would be competent enough to replace petrobased commercially available coatings.

## 2. Materials

Canola oil (Afia International Company, Jeddah, Saudi Arabia), Lactic acid [LA], sodium metal, methanol, toluene (BDH Chemical, Poole, England), Diethanolamine (Loba chemie, India), toluene-2,4-diisocyanate [TDI] (Acros Organic, NJ, USA) and fumed silica [FS] (Sigma-Aldrich, St. Louis, MI, USA) were used as received.

## 3. Instrumentation

The structural elucidation was performed by spectral analysis. FTIR was carried out on FTIR spectrophotometer (Spectrum 100, Perkin Elmer Cetus Instrument, Norwalk, CT, USA). NMR spectrum (^1^H NMR and ^13^C-NMR) was recorded (on JEOL DPX400MHz, Japan) using deuterated Chloroform (CDCl_3_) as a solvent and tetramethylsilane (TMS) as an internal standard. Thermogravimetric analysis [TGA] (on Mettler Toledo AG, Analytical CH-8603, Schwerzenbach, Switzerland) was accomplished to study the thermal stability of synthesized material. TGA was performed in a nitrogen atmosphere and at a heating rate of 10 °C/min. Field Emission Scanning Electron Microscopy (FE SEM, JSM 7600F, JEOL, Tokyo, Japan) and Energy dispersive X-ray spectroscopy (EDX, Oxford, UK) studies revealed the morphology of coating material. Acid value (ASTM D555–61), scratch hardness (BS 3900), impact test (IS 101 part 5 s^−1^, 1988), flexibility/bending test (ASTM D3281-84), gloss (by Gloss meter, Model: KSJ MG6-F1, KSJ Photoelectrical Instruments Co., Ltd. Quanzhou, China), and thickness measurements (ASTM D 1186-B) were performed by standard methods. The hydrophobicity of coatings was evaluated by carrying out the contact angle measurements by aCAM200 Attention goniometer, using deionized water and water drops were allowed to fall onto the substrate, i.e., the coated panel. The angle measurements were done in triplicate.

Corrosion test specimens were attended as working electrode. An exposed surface area of 1.0 cm^2^ was fixed by PortHoles electrochemical sample mask, with Pt electrode as counter electrode, and 3 M KCl filled Ag electrode as a reference electrode (Auto lab Potentiostat/galvanostat, PGSTAT204-FRA32, with NOVA 2.1 software; Metrohom Autolab B.V. Kanaalweg 29-G, 3526 KM, Utrecht, Switzerland). For reproducibility purposes, each corrosion test was performed in triplicate. 

## 4. Synthesis

### 4.1. Synthesis of Canola Diol Fatty Amide (CFA)

CFA was prepared according to our previously published article using vegetable oil and diethanolamine in the presence of sodium methoxide catalyst [44]. The synthesized product was washed using diethyl ether and 15% sodium chloride solution and the structure of CFA was confirmed by FTIR analysis.

### 4.2. Synthesis of Canola Oil based Poly(ester–ether–amide–urethane) (CPEEUA) and CPEEUA/FS Nanocomposite 

**Scheme 1:**
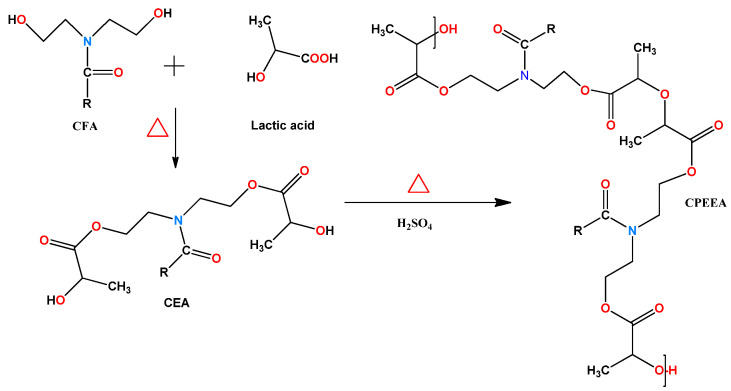
Synthesis of Canola oil based poly(ester–ether) amide [CPEEA].

The synthesis of CPEEUA was carried out by the following steps:

Step 1: Synthesis of CANO ester amide [CEA]: CFA (0.05 mol) and LA (0.10 mol) were placed in a four-necked round-bottomed flask fitted with dean stark trap, a nitrogen inlet tube, and thermometer, and heated at 80 °C for 3h, over a magnetic stirrer. The reaction temperature was increased to 120 °C and maintained at this temperature for another 2 h until desired (low) acid value was obtained (Scheme 1). The reaction was monitored by thin layer chromatography (TLC) and FTIR spectrum was recorded.

Step 2: Synthesis of poly(ester–ether) amide (CPEEA).

CEA was taken in a reaction flask, and to it was added 25 mL toluene (for azeotropic distillation) and 1 mL H_2_SO_4_ (1:1 *v*/*v* diluted with water) dropwise. Subsequently, the temperature was increased to 140 °C, and stirring was continued at this temperature, until FTIR supported the formation of ether linkages, i.e., the formation of CPEEA (Scheme 1). The reaction was monitored by TLC and recording FTIR spectra at regular intervals of time.

**Scheme 2:**
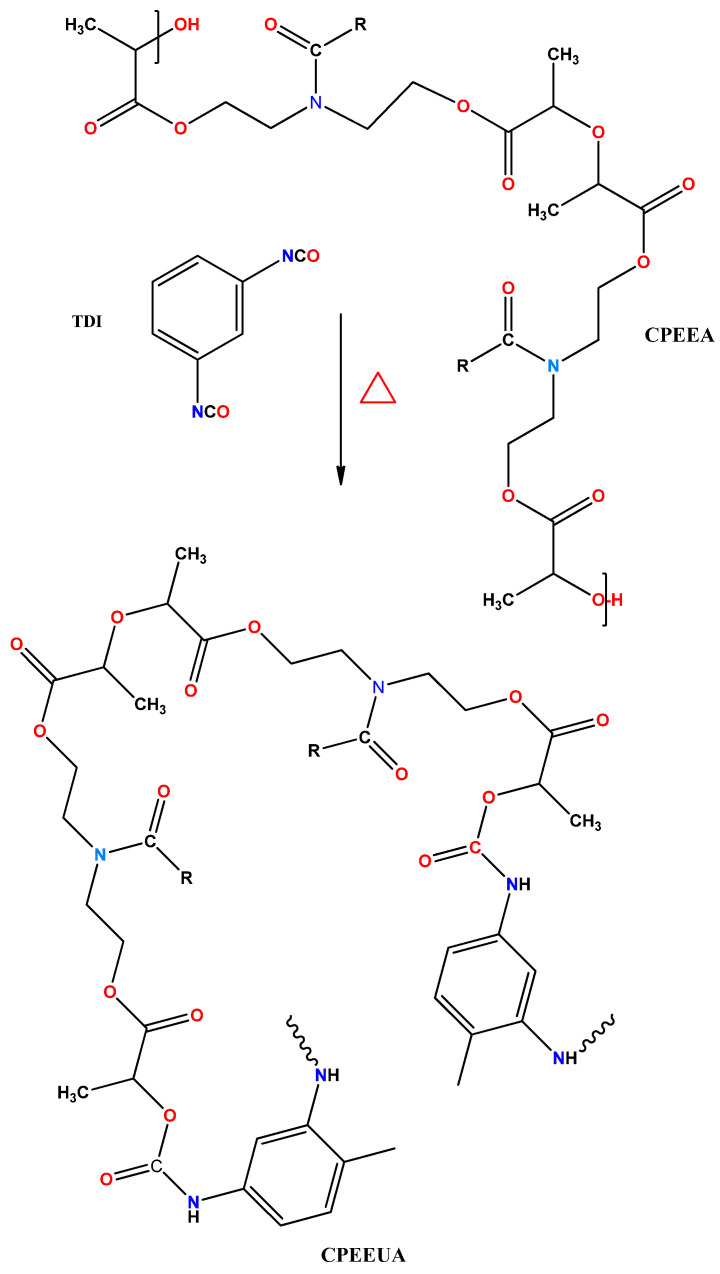
Synthesis of Canola oil poly (ester–ether–amide–urethane) (CPEEUA).

Step 3: Synthesis of CPEEA/fumed silica nanocomposite (CPEEA/FS): FS (2% *w*/*w* on the weight of CFA) was added with gradual mixing to the calculated amount of CPEEA, followed by the addition of TDI (35, 40, 45% *w*/*w* on the weight of CFA), dropwise under continuous stirring. After the complete addition of TDI, the reaction temperature was increased to 60 °C, and the reaction was continued until the FTIR spectrum indicated the formation of polyurethane (Scheme 2).

Different CPEEUA/FS nanocomposites were prepared by adding TDI in different weight percentages (35, 40, 45% *w*/*w* on the weight of CFA), in similar experimental set-up, to obtain CPEEUA/FS-35, CPEEUA/FS-40 and CPEEUA/FS-45 polyurethane nanocomposites (35, 40 and 45 indicate the percent loading of TDI). The reaction was monitored by thin layer chromatography and FTIR.

A similar reaction was also accomplished omitting the addition of FS, producing plain CFA based polyurethane, CPEEUA.

## 5. Preparation of CPEEUA/FS Nanocomposite Coatings

Carbon steel [CS] strips in two standard sizes (70 mm × 25 mm × 1 mm and 25 mm × 25 mm × 1 m) and composition (Fe, 99.51%, Mn, 0.34%, C, 0.10% and P, 0.05%) were first polished with silicon carbide paper of different grades, then washed with double distilled water, followed by methanol and acetone for degreasing, and next dried at room temperature. These cleaned CS strips were then coated with 40% (*w*/*v*% in toluene) solutions of CPEEUA and CPEEUA/FS by brush and the coated panels were left to dry at room temperature for two weeks for complete curing/drying. The dried coated panels were then subjected to physico-mechanical and corrosion tests by standard methods.

## 6. Results and Discussion

CFA was prepared by base catalysed amidation reaction of CANO [37,38,39]. CPEEAU/FS nanocomposite was prepared using CFA, LA, FS, and TDI as raw materials, by esterification, etherification, and urethanation. The aliquots of samples were withdrawn to govern the progress of the reaction by TLC and FTIR (which gave a clue about the proposed structure of the end product, based upon the spots in the TLC plate and appearance or disappearance of corresponding functional groups’ absorption bands in the FTIR). After indication from FTIR, the samples were then subjected to NMR spectral analysis for confirmation of respective structures. The synthesized CPEEAU/FS was then used for preparation of coatings.

LA bears both hydroxyl and carboxyl functional groups and thus introduces both ether and ester linkages in CANO polyurethane [20,21,22]. CPEEAU is cured at room temperature due to the presence of urethane linkages and the double bonds of the triglyceride chains that undergo auto-oxidation.

### 6.1. Solubility

CPEEAU/FS were soluble in toluene, xylene, chloroform, carbon tetra chloride, dimethyl formamide, dimethyl sulphoxide, ethyl methyl ketone, acetone, acrylonitrile, ethyl acetone, tetrahydrofuron, benzene, pyridine, cyclohexane, 1–4 dioxane, sparingly soluble in dichloro methane, di ethyl ether, ethanol, methanol, formamide, n-butanol, n-propanol, benzyl alcohol, and insoluble in n-hexane and distilled water.

### 6.2. Spectral Analysis

**Figure 1:**
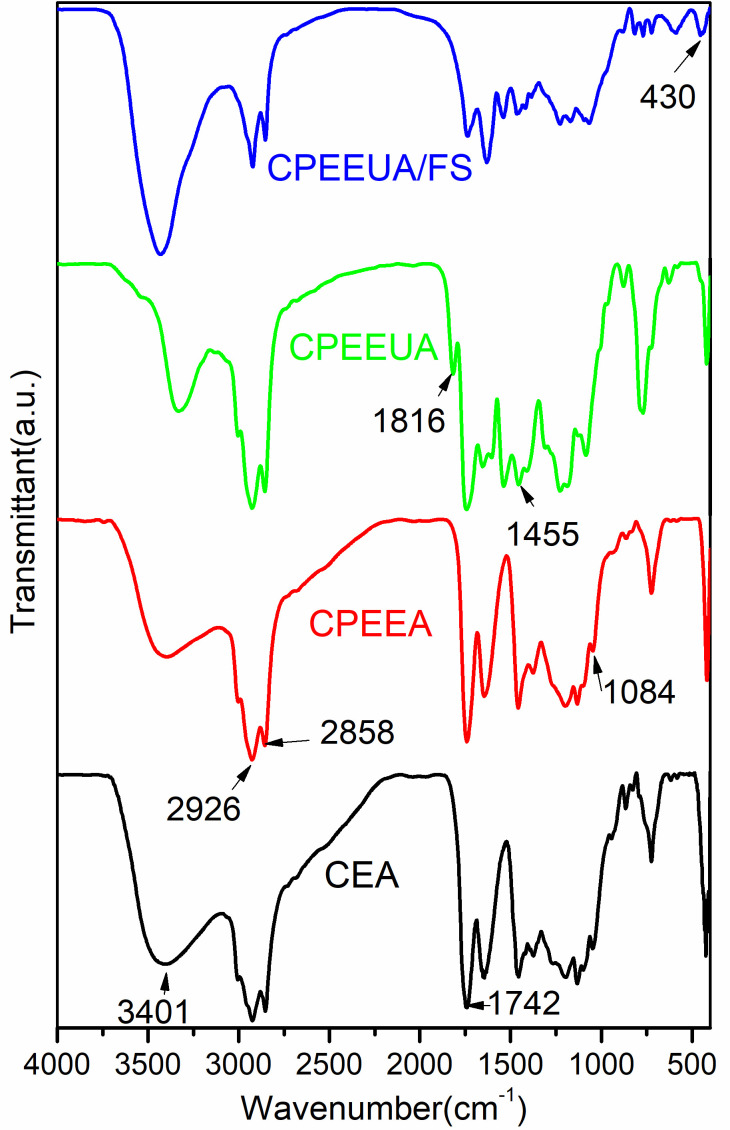
FTIR spectra of CEA, CPEEA, CPEEUA, and CPEEUA/FS for functional group determination.

**CEA, FTIR(cm^−1^):** 3401 (–OH), 3004 (H-C=C-str), 2924 and 2853 (–CH_2_, asymmetrical and symmetrical), 1742 (–C=O, ester), 1645 (–C=O, amide), 1455 (–C–N), 1372, 770 (–CH_2_, CH_3_, bending vibrations), 1196, 1132, 1099 (–C–C(=O) –O–C–), 1047 (–C–OH) (Figure 1).

**CPEEA, FTIR (cm^−1^):** 3396 (–OH), 3002 (H–C=C-str), 2926 and 2858 (–CH_2_, asymmetrical and symmetrical), 1740 (–C=O, ester), 1644 (–C=O, amide), 1457 (–C–N), 1375, 768 (–CH_2_, CH_3_ bending vibrations), 1197, 1132, 1099 (–C–C(=O) –O–C–), 1046 (–C–OH), 1070 (–C–O–C–, ether) (Figure 1). CPEEA shows suppressed –OH bands relative to those in CEA, as well as the appearance of bands at 1070 that support the consumption of –OH groups and formation of ether linkages.

**CPEEUA, FTIR (cm^−1^):** 3330 (–NH, urethane), 3003 (H–C=C–str), 2925 and 2856 (–CH_2_ asymmetrical and symmetrical), 1816 (–C=O, urethane), 1742 (–C=O, ester), 1652 (–C=O amide), 1455 (–C–N), 1226, 1188, 1099 (–C–C(=O) –O–C–), 1084 (–C–O–C–, ether), 1537, 877, 770–750 (aromatic urethane) (Figure 1).

**CPEEUA/FS FTIR(cm^−1^):** 3332 (–NH, urethane), 3004 (H–C=C– str), 2924 and 2864 (–CH_2_, asymmetrical and symmetrical), 1815(–C=O, urethane), 1743 (–C=O, ester), 1657 (–C=O, amide), 1454 (–C–N), 1224, 1133, 1094 (–C–C(=O)–O–C–), 1084, 1094 (–C–O–C–, ether & Si–O–Si, asymm str), 1537, 875, 770–750 (aromatic urethane), 422–440 (Si–O–Si, bending); 818 (Si–O–Si, sym str), 950 (Si–OH) (Figure 1) [29,45]. CPEEUA/FS shows absorption bands as in CPEEUA; however, it also shows additional absorption bands that support the inclusion of FS in the polymer chains.

**Figure 2:**
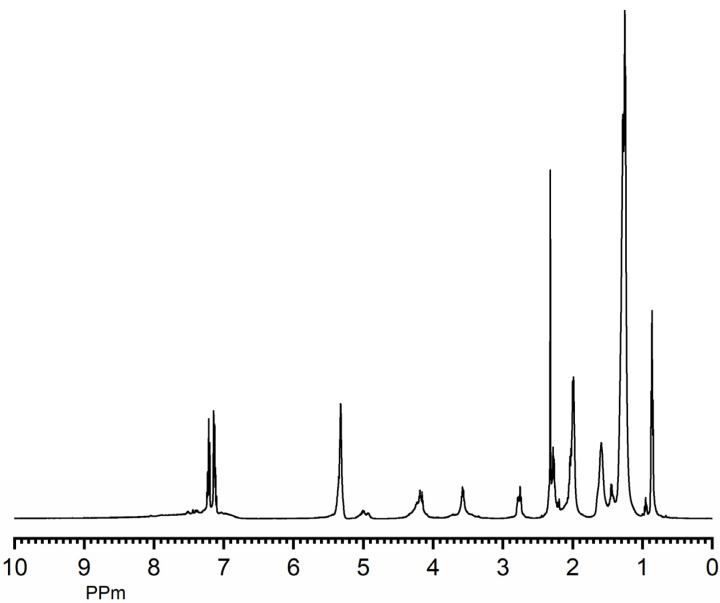
**^1^**HNMR spectrum of CPEEUA for structure elucidation.

**CPEEUA, ^1^H NMR (CDCl_3_, *δ* ppm):** 0.850–0.875 (–C**H**_3_, fatty amide chain), 1.438 (–C**H**_3_, lactic acid), 1.244–1.452(–C**H**_2_, chain), 1.591 (–CO–CH_2_–C**H**_2_), 1.988 (–C**H**_2_ attached to double bond), 2.26 (–CO–C**H**_2_–CH_2_), 2.741–2.787 (–C**H**_2_, flanked by double bonds), 3.565–4.159 (O–C**H**_2_–CH_2_–N< and –O–CH_2_–C**H**_2_–N<), 4.337 (–C**H**–, lactic acid), 5.324–5.352 (–C**H**=C**H**–), 2.321 (–C**H**_3_ of urethane), 7.101–7.241 (Aromatic urethane), 7.407–7.513 (–N**H** urethane) (Figure 2).

**Figure 3:**
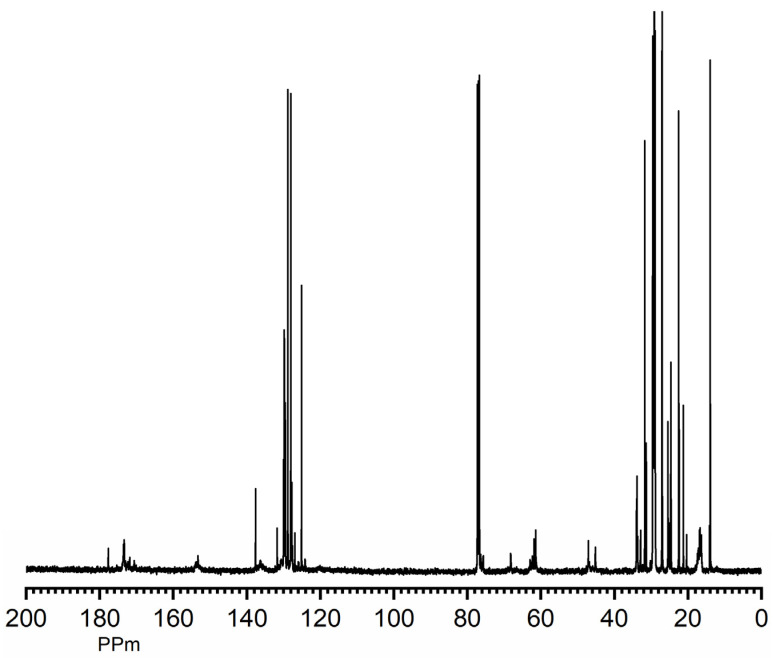
**^13^**CNMR spectrum of CPEEUA for structure elucidation.

**CPEEUA, ^13^C NMR (CDCl_3_, *δ* ppm):** 13.9–14.1 (–**C**H_3,_ fatty amide chain), 16.365–17.452 (–CH_3_, TDI), 20.390 (–CH_3_, lactic acid), 21.268–25.474 (–**C**H_2_–**C**H_3_), 28.822–28.946 (–**C**H_2_ attached CO–), 29.032–29.604 (chain –**C**H_2_), 31.359–31.750 (–**C**H_2_ attached to double bond), 47.069 (–O–CH_2_–**C**H_2_–N–), 61.424–61.825 (–O–**C**H_2_–CH_2_–N–), 68.196 (–**C**H, lactic acid) 124.157–137.626 (double bond and aromatic carbons), 153.268 (–C=O urethane), 171.840–177.639 (–C=O amide, ester). Spectral analyses thus confirmed the structure of CPEEUA (Figure 3) [37,38,39].

### 6.3. Physico-Mechanical Test of Coatings

The thickness of coatings was found to be 95 micron to 105 micron. Scratch hardness increased from 2.0 kg to 2.2 kg to 2.5 kg, and then decreased to 2.4 kg with increased urethane and aromatic content in CPEEUA-40, CPEEUA/FS-35, CPEEUA/FS-40 and CPEEUA/FS-45, indicating that at 40% *w*/*w* loading of TDI in CPEEUA/FS-40, the best scratch hardness resistance could be achieved. Good impact resistance (150lb/inch) and bending ability was also achieved in CPEEUA/FS-40 due to good adhesion of coated films to the substrate and crosslinked polymer chains. Beyond 40% *w*/*w* addition of TDI, increased aromatic content and excessive crosslinking conferred by urethanation reaction caused brittleness and the coating properties were deteriorated. Thus, CPEEUA-40 and CPEEUA/FS-40 were selected as the study sample to perform corrosion resistance, surface wettability, and thermal stability tests. 

### 6.4. Surface Wettability 

**Figure 4:**
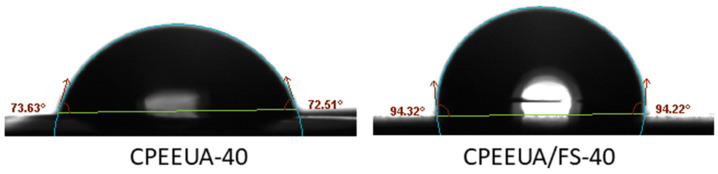
Contact angle of CPEEUA-40 and CPEEUA/FS-40 to evaluate hydrophobicity.

Surface wettability of CPEEUA-40 and CPEEUA/FS-40 was studied by contact angle (Figure 4) measurement. CPEEUA-40 and CPEEUA/FS-40 show water contact angle values of 73° ± 2° and 94° ± 2°, respectively. These values are indicative of hydrophilic nature of the former and hydrophobic behavior of the latter due to the inclusion of FS. The homogenous dispersion of FS in CPEEUA/FS-40 matrix fills the voids within the matrix and increases the roughness of its film surface, thus improving the barrier properties of the nanocomposite coating [25,46,47].

### 6.5. Morphology (SEM/EDX)

**Figure 5:**
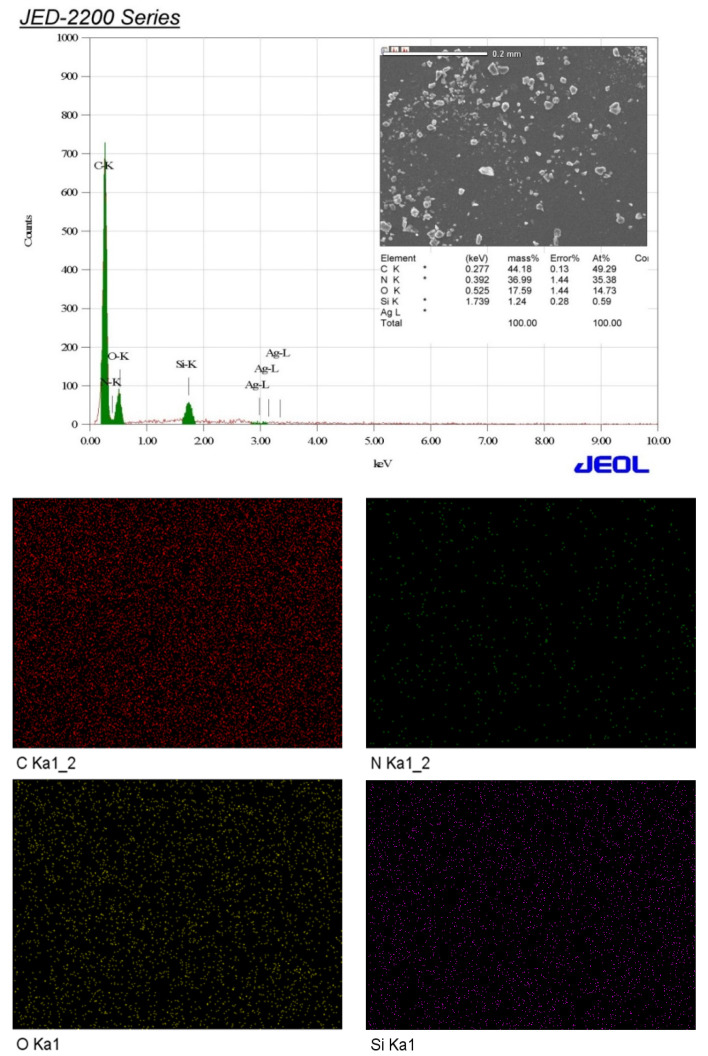
SEM -EDX with elemental mapping of CPEEUA/FS-40 to assess morphology.

Figure 5 shows the distribution of FS in CPEEUA/FS-40 matrix. EDX and elemental analysis confirmed the dispersion of FS in the CPEEUA matrix. The energy peaks of FS (Si), C, N, and O are evident and confirm the presence of FS in CPEEUA matrix, with their respective content of 44.18, 36.99 and 17.59. The wt% of SiO_2_ in CPEEUA/FS-40 matrix was found to be 1.24%, which is consistent with its inclusion (2%) in the CPEEUA/FS-40 matrix. All elements are spread throughout the film and impurities are absent, exhibiting the uniform and homogenous nature of the film. EDX and elemental mapping analysis confirmed the dispersion of FS in the polymer matrix.

### 6.6. Thermal Stability

**Figure 6:**
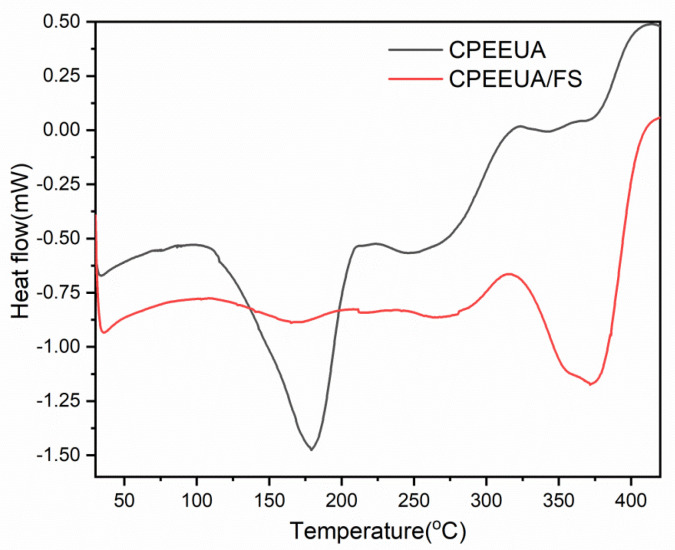
DSC of CPEEUA-40, CPEEUA/FS-40.

DSC thermogram of CPEEUA-40 (Figure 6) shows that the first endothermic event starts from 108 °C to 209 °C, centered at 178 °C. While this endothermic event is insignificant in CPEEUA/FS-40. This first endothermic event may be correlated to the loss of moisture. The second endothermic event extends from 226 °C to 321 °C in CPEEUA-40 and CPEEUA/FS-40. The third significant and broad endotherms appear from 322 °C to 412 °C, respectively, in CPEEUA-40 and CPEEUA/FS-40, which can be attributed to the configurational changes in polymer backbone due to subsequent thermal degradation stages as evident in TGA and DTG.

**Figure 7:**
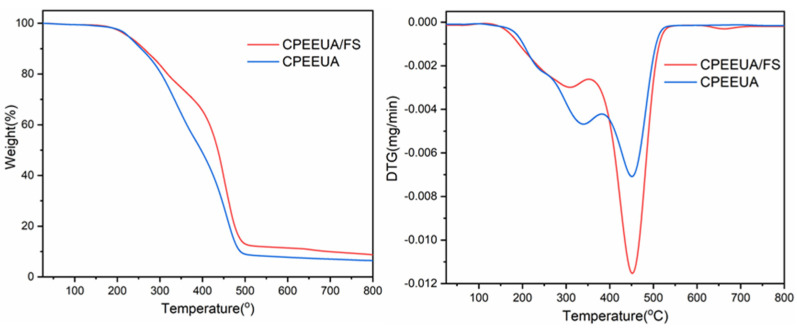
TGA and DTG of CPEEUA-40 and CPEEUA/FS-40 to compare their thermal stability.

TGA thermograms (Figure 7) show the first 10 wt% weight loss up to 250 °C, which can be attributed to the loss of moisture; both CPEEUA-40 and CPPEUA/FS-40 exhibit the same degradation pattern as evident from TGA and DTG (Figure 7). Beyond this temperature, the degradation can be attributed to the onset of decomposition of the urethane bonds, proceeded by subsequent degradation of other moieties of polymer backbone, under the effect of rising temperature. A significant variation is observed in the degradation pattern of CPEEUA/FS-40 from TGA and DTG thermogram; this showcases its improved thermal stability relative to CPEEUA-40. 20wt%, 30wt%, 40wt%, 50wt%, 60wt%, and 70 wt% losses are observed at 300 °C, 333 °C, 363 °C, 397 °C, 425 °C, 445 °C, and 316 °C, 376 °C, 417 °C, 434 °C, 449 °C, 459 °C, in CPEEUA-40 and CPEEUA/FS-40, respectively. The remarkable variation in thermal degradation temperatures, for each wt% loss, in CPEEA-40 and CPEEUA/FS-40 highlights good thermal stability of the nanocomposite, due to fine and homogenous dispersion of FS as well as good interfacial interactions with the matrix. CPEEUA-40 displays a somewhat single-step degradation pattern contrary to CPEEUA/FS-40 which exhibits a notable two-step degradation pattern, covering 90 wt% loss. TGA indicates that these coatings can be safely used up to 250 °C.

### 6.7. Potentiodynamic Polarization

**Figure 8:**
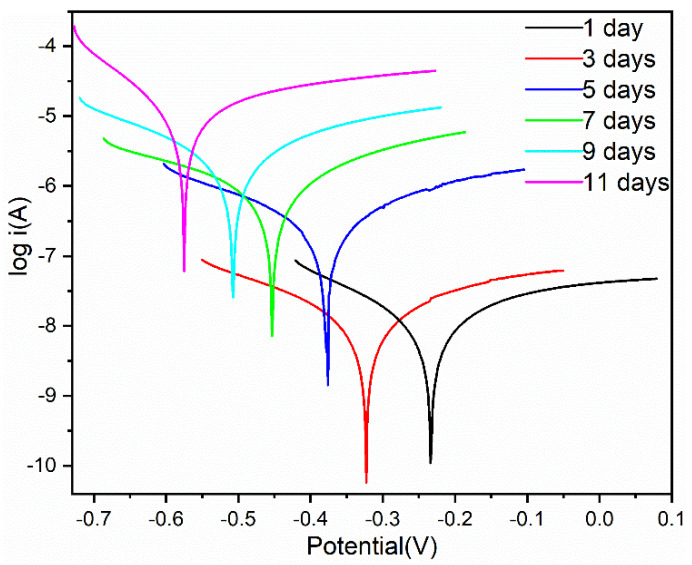
Tafel spectrum of CPEEUA/FS conducted in 3.5 wt% NaCl solution (corrosive medium) as a function of exposure times.

Tafel polarization curves (Figure 8) for CPEEUA/FS-40 coating were conducted in a 3.5 wt% NaCl solution chosen as a corrosive medium. The data pertaining to corrosion rate (CR), corrosion potential (Ecorr), corrosion current density (Icorr), and polarization resistance are tabulated (Table 1) by the tafel extrapolation method. CPEEUA/FS-40 showed good corrosion protection for MS. However, it is clear from the table that the corrosion potential of CPEEUA/FS-40 coatings pointedly decreased and corrosion current density increased with increased immersion time in the said corrosive medium. These results indicate that CPEEUA/FS-40 can act as protective layer of MS and improve the corrosion resistance performance up to 7 days, then reduce the coating performance due to the formation of some pores in coatings, resulting in contact of corrosive ions to substrate during the long immersion time (11 days) and corrosion potential of CPEEUA/FS-40 coatings also shift towards the more negative potential during these immersion times [32]. 

### 6.8. Electrochemical Impedance Spectroscopy

**Figure 9:**
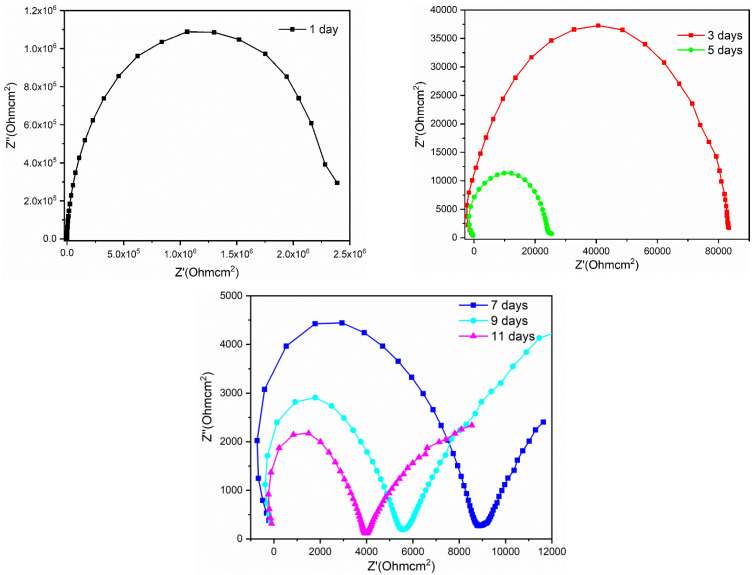
EIS spectrum of CPEEUA/FS conducted in 3.5 wt% NaCl solution (corrosive medium) as a function of exposure times.

In Figure 9, the corrosion protection performance of CPEEUA/FS-40 was investigated by EIS during 1, 3, 5, 7, 9, and 11 days of immersion of nanocomposite coated metal strips in a 3.5 wt% NaCl medium, as a function of exposure times. Table 2 distinctly revealed that the Cc increased with the immersion time while Rct decreased. CPEEUA/FS-40 throughout immersion time (5 days) showed only one capacitive loop. As immersion time increased, capacitive loop decreased, as shown in the Warburg diffusion (Figure 9). The impedance data (Table 2) was observed at 10^6^ Ω, on day 1, which reduced to 10^4^ Ω after increasing the immersion time to 3 days. The impedance value seems to be affected by the immersion period; although for first 5 days little change in impedance was observed for CPEEUA/FS-40 coatings. On the eleventh day, comparatively more significant loss in impedance was observed. Thus, the CPEEUA/FS-40 coating obstructs the charge transfer reactions taking place at the metal strip surface and the medium to which it is exposed, up to 7 days. This is expected as with continuous immersion (11 days), CPEEUA/FS-40 coating surface would infuse some corrosive ions through the coating itself. 

## 7. Conclusions

The manuscript described the preparation of a poly (ester–ether–urethane) amide from Canola oil. CPEEUA was further strengthened by including fumed silica as a nanofiller, yielding nanocomposite, which was further applied as a corrosion protective coating material. The coatings showed good physico-mechanical and corrosion resistance against a 3.5 wt% NaCl solution. The approach paved path for utilization of vegetable oils by simple, single-pot derivatization method, to be applied as organic coatings and nanocomposite coatings.

## Figures and Tables

**Table 1:** Tafel parameters of CPEEUA/FS-40 nanocomposite.

Immersion Time (Days)	Ba (V/dec)	Bc (V/dec)	Corrosion Potential E_corr_ (V)	Corrosion Current Density, I_corr_ (A/cm^2^)	Corrosion Rate (mm/Year)	Polarization Resistance (Ω·cm^2^)
1	0.3158	0.2122	−0.2335	9.9654 × 10^−9^	1.1579 × 10^−4^	5.5318 × 10^6^
3	0.6418	0.2853	−0.3229	2.0314 × 10^−8^	2.3407 × 10^−4^	4.2588 × 10^6^
5	0.5548	0.2487	−0.3757	2.4331 × 10^−7^	2.827 × 10^−3^	2.1798 × 10^5^
7	0.3430	0.2980	−0.4531	9.0153 × 10^−7^	1.047 × 10^−3^	7.6825 × 10^4^
9	0.2502	0.3043	−0.5072	2.2913 × 10^−6^	2.662 × 10^−2^	2.6021 × 10^4^
11	0.1243	0.2707	−0.5750	7.9805 × 10^−6^	9.273 × 10^−2^	4.637 × 10^3^

**Table 2:** EIS parameters of CPEEUA/FS-40 nanocomposite.

Immersion Time (Days)	Solution Resistance, Rs (Ω)	Charge Transfer Resistance, Rct(Ω)	Coating Capacitance Cc (Pf)	W (µmho × s^−1^)
1	604	2.210 × 10^6^	146	-
3	554	8.100 × 10^4^	178	-
5	490	2.450 × 10^4^	198	-
7	351	8.881 × 10^3^	246	98.2
9	257	5.630 × 10^3^	282	142
11	193	4.090 × 10^3^	343	214

## Data Availability

The data presented in this study are available on request from the corresponding author.
